# Molecular characterisation of *Vibrio cholerae *O1 strains carrying an SXT/R391-like element from cholera outbreaks in Kenya: 1994-2007

**DOI:** 10.1186/1471-2180-9-275

**Published:** 2009-12-29

**Authors:** John N Kiiru, Suleiman M Saidi, Bruno M Goddeeris, Njeri C Wamae, Patrick Butaye, Samuel M Kariuki

**Affiliations:** 1 Centre for Microbiology Research, Kenya Medical Research Institute, PO Box 43640, Nairobi, Kenya; 2 Department of Biosystems, Faculty of Bio-Science Engineering, Katholieke Universiteit Leuven, Kasteelpark Arenberg 30, B-3001, Heverlee, Belgium; 3 Veterinary and Agrochemical Research Centre, Groeselenberg 99, B-1180, Ukkel, Belgium; 4 Department of Virology, Parasitology and Immunology, Faculty of Veterinary Medicine, University of Ghent, Salisburylaan 133, 9820, Merelbeke, Belgium; 5 Department of Pathology, Bacteriology and Poultry Diseases, Faculty of Veterinary Medicine University of Ghent Salisburylaan 133,9820, Merelbeke, Belgium

## Abstract

**Background:**

Over the last decade, cholera outbreaks in parts of Kenya have become common. Although a number of recent studies describe the epidemiology of cholera in Kenya, there is paucity of information concerning the diversity and occurrence of mobile genetic elements in *Vibrio cholerae *strains implicated in these outbreaks. A total of 65 *Vibrio cholerae *O1 El Tor serotype Inaba isolated between 1994 and 2007 from various outbreaks in Kenya were investigated for mobile genetic elements including integrons, transposons, the integrating conjugative elements (ICEs), conjugative plasmids and for their genotypic relatedness.

**Results:**

All the strains were haemolytic on 5% sheep blood and positive for the *Vibrio cholerae *El Tor-specific haemolysin toxin gene (*hylA*) by PCR. They all contained *strB, sulII, floR *and the *dfrA1 *genes encoding resistance to streptomycin, sulfamethoxazole, chloramphenicol and trimethoprim respectively. These genes, together with an ICE belonging to the SXT/R391 family were transferable to the rifampicin-resistant *E. coli *C600 *en bloc*. All the strains were negative for integron class 1, 2 and 3 and for transposase gene of transposon *Tn*7 but were positive for integron class 4 and the *trpM *gene of transposon *Tn*21. No plasmids were isolated from any of the 65 strains. All the strains were also positive for all *V. cholera *El Tor pathogenic genes except the NAG- specific heat-stable toxin (*st*) gene. None of the strains were positive for virulence genes associated with the *V. cholerae *classical biotype. All the strains were positive for El Tor-specific CTXphi bacteriophage *rstrR *repressor gene (*CTX*^ET^*Φ*) but negative for the Classical, Calcutta, and the Environmental repressor types. Pulse Field Gel Electrophoresis (PFGE) showed that regardless of the year of isolation, all the strains bearing the SXT element were clonally related.

**Conclusions:**

This study demonstrates that the *V. cholerae *O1 strains carrying an SXT/R391-like element implicated in recent cholera outbreaks in Kenya has not changed significantly between 1994 and 2007 and are clonally related.

## Background

Since 1971, Kenya has suffered many outbreaks of cholera. From 1974 to 1989, outbreaks were reported every year with an average case fatality rate of 3.6% [1]. For instance, the 1994 cholera outbreaks started in Kwale on the Kenyan coastline and affected 3 districts in the Coast province; Kwale, Mombasa and Taita-Taveta. Between 1997 and 1999, more than 33,400 notified cases of cholera were reported in Kenya, representing 10% of all cholera cases reported from the African continent during this period [2,3]. From 2000 to 2006, cases ranging from 816 to 1,157 were reported each year except for 2002, in which 291 cases were reported [1]. More cases have been reported locally since 2005 [4] and the recent outbreak in 2007 had a case fatality of up to 5.6% [1].

Strains belonging to serotype Ogawa were frequently isolated from cholera outbreaks in Kenya in the 1970s through 1990s and many isolates from this period were known to exhibit resistance to a diverse range of antibiotics including chloramphenicol, tetracycline, ampicillin, streptomycin, sulfonamides and doxycycline [5,6]. In the recent past however, serotype Inaba has emerged as the main cause of epidemics in Kenya and these isolates are frequently not susceptible to chloramphenicol, streptomycin, sulphonamides, sulfamethoxazole and trimethoprim (Chl-Str-Sul-Trim). A mobile genetic element (MGE) belonging to the SXT family of ICEs, was shown to confer this phenotype in the strains isolated during the 1998-1999 period [7]. It is however unknown if strains isolated prior to and after this period harbour this element.

The integrase gene of the SXT family of ICEs is highly related to the one found in the R391 element [8] and is also closely related to the one found in conjugative transposons and bacteriophages [9]. Upon conjugation, SXT/R391-like ICEs integrate into the *prfC*, a gene found on the large *V. cholerae *chromosome [10]. In the SXT-like elements, genes encoding antibiotic resistance are integrated into the *rumB *thus interrupting the *rumAB *operon while in the R391, this operon is not interrupted [11,12]. An SXT element, SXT^MO10^, was detected in *V. cholerae *from a O139 biotype strain from Madras, India and is known to confer the Chl-Str-Sul-Trim phenotype [12]. This element is related to ICEVchInd1 found in O139 and El Tor strains [12,13]. Burrus *et al*. (2006) gave a detailed review of the ICE biology and classification [14]. We investigated 65 strains exhibiting the Chl-Str-Sul-Trim phenotype isolated from various parts of Kenya from 1994 through 2007 for the presence of SXT/R391-like elements and for evidence of integration of the element into the host chromosome.

We also determined the diversity of *rstR *genes encoding the cholera CTX-prophage repressor from the 65 strains isolated from the same period. Although most sequences in the CTXΦ-prophage genomes are similar in the El Tor and Classical biotypes strains, the *rstR *specific to the biotype-specific prophages differ. The El Tor and Classical biotype strains carry the CTX^ET^Φ and the CTX^Class^Φ repressor types, respectively [15,16] while the CTX^Calc^Φ and CTX^Env^Φ encode the Calcutta and Environmental *rstR *types, respectively [17,18]. Strains known as the Matlab variants belonging to the El Tor biotype but harbouring the CTX^class^Φ prophage have been isolated in Bangladesh [19], India [20] and Mozambique [21]. Three classes of multiresistant (MR) integrons (class 1, 2 and 3) are known to harbour genes encoding resistance to antibiotics [22-24]. Integron class 4 is commonly found in *V. cholerae *and is referred to as a super integron (SI). Although integrons are not capable of self-transposition, they are known to associate with insertion sequences (ISs), transposons, and/or conjugative plasmids which serve as vehicles for the intra- and interspecies transmission of genetic material [24]. Like the R391 ICE elements, transposon *Tn*21 and its relatives frequently harbour genes conferring resistance to mercury and antibiotics-containing integrons that integrate into the left arm adjacent to the *tnpM *gene [25]. Transposon *Tn*7 is also known to associate with integron class 2 and is therefore an important MGE [26]. We therefore also analysed the 65 strains for the presence of integron classes 1, 2, 3 and 4, conjugative plasmids, the *tnpM *gene of transposon *Tn*21 and the transposase of *Tn*7 transposon.

## Methods

### Sources of *Vibrio cholerae *strains

**Figure 1:**
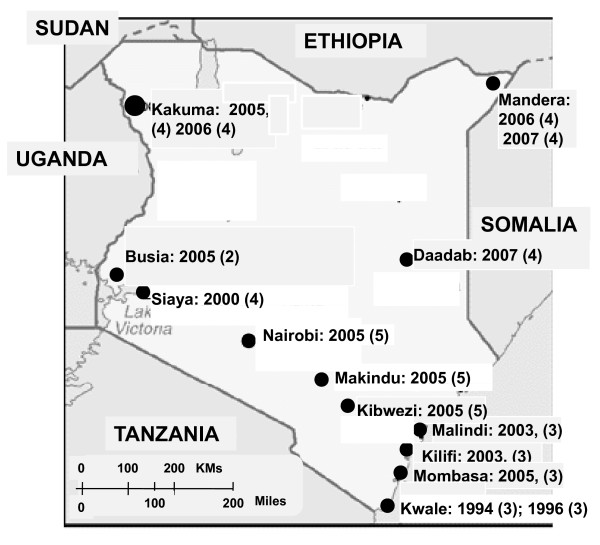
**Sources of *V. cholera *strains used for this study**. The geographic locations from which the isolates were obtained are indicated using a black dot. The number of the strains and the year of isolations are also indicated. All the strains from various regions regardless of the year of isolation had an identical profile for antibiotic susceptibility profiles and for genes associated with resistance and virulence in *V. cholerae*.

Strains that were included in this study were obtained from distinct outbreaks occurring in different parts of Kenya between 1994 and 2007 as indicated in figure 1. For consistency, a distinct outbreak was defined as a gap of at least 2 months between the last known cholera case and a report of a new case in the same location. Archived isolates were initially subcultured on thiosulphate citrate bile salts sucrose agar (TCBS) and confirmation of strain identity was done by serology using polyvalent, anti-Ogawa, and anti-Inaba antisera (Denka Seiken, Tokyo, Japan). Haemolysis test was done by growing *V. cholerae *on 5% sheep blood nutrient agar plates incubated at 37°C overnight.

### Antimicrobial susceptibility testing

Antimicrobial susceptibility tests were performed using commercial discs following manufacturer's instructions (Cypress diagnostics, Langdorp, Belgium). Susceptibility to β-lactam antibiotics was tested using ampicillin (10 μg) while susceptibility to cephalosporins was determined using cefixime (30 μg), cefotaxime (30 μg), cefepime (30 μg) cefoxitin (30 ug), cefuroxime (30 ug), ceftriaxone (30 ug), and ceftazidime (30 ug). Ciprofloxacin (5 μg), norfloxacin (10 μg) and nalidixic acid (30 μg) were used for testing susceptibility to the quinolones. Aztreonam (30 μg), a monobactam antibiotic, was also included in the assay. Aminoglycosides used in susceptibility tests included kanamycin (30 μg), amikacin (30 μg), streptomycin (30 μg), gentamicin (10 μg), neomycin (30 μg), and tobramycin (10 μg). Tetracycline antibiotics included minocycline (30 μg), doxycycline (30 μg) and tetracycline (30 μg). Other antibiotics included chloramphenicol (30 μg), furazolidone (50 μg), rifampicin (30 μg) and nitrofurantoin (30 μg). Sulphamethoxazole (25 μg), trimethoprim (5.2 μg) and sulphonamides (300 μg) were also tested. β-lactam and β-lactamase inhibitor combinations included augmentin (comprising 20 μg amoxicillin and 10 μg clavulanic acid) and a combination of piperacillin (100 μg) and tazobactam (10 μg). *E. coli *ATCC 25922 was used as a control for bacterial growth and potency of antibiotic discs. Susceptibility tests were interpreted using the Clinical and Laboratory Standards Institute guidelines [27].

### PCR amplification

DNA used as template for PCR reactions was prepared from overnight L-broth cultures incubated at 37°C. Bacterial cells were harvested by centrifugation and re-suspended in 1 ml 10 mM Tris/HCl (pH8·0) containing 1 mM EDTA. Template DNA was obtained by boiling for 10 min and separated by centrifugation at 12,000 × g for 3 min and then stored at -20°C until analysed. PCR was carried out in 50 μl reaction volumes containing 5 μl 10× concentrated PCR buffer [100 mM Tris/HCl (pH8·3), 500 mM KCl, 15 mM MgCl_2_], 5 μl (10 pmol μl^-1^) each of primer, 4 μl dNTP mix (2·5 mM each dNTP), 0.25 μl (5 U μl^-1^) *Taq *DNA polymerase, 5 μl of template DNA and 25.75 μl sterilized distilled water. All PCR assays were performed using an automated thermal cycler (GeneAmp PCR System 9700; Applied Biosystems). PCR products were analysed by electrophoresis in 1.5% agarose gels, stained with ethidium bromide, visualized under UV light and recorded with the aid of a gel documentation system (Bio-Rad Laboratories, Hercules, Ca, USA)

### Conjugation experiments and PCR screening for antibiotic resistance genes

The mating assays were carried using the rifampicin-resistant *E. coli *C600 strain as the recipient. Conjugations were carried out at 37°C for 8 hr without shaking. Transconjugants were selected on Mueller-Hinton agar plates (Oxoid Ltd; Basingstoke, Hampshire, England) containing trimethoprim (5.2 μg/ml) and rifampicin 30 μg/ml. In order to confirm that the antibiotic resistance gene markers were transferred during conjugation, the donor and transconjugants were analysed using PCR methods. Screening of the *sulII *gene encoding resistance to sulfamethoxazole, *dfrA1 *encoding resistance to trimethoprim and *strB *encoding resistance to streptomycin was done as described previously by Ramachandran *et al*. [28] while detection of the *floR *conferring resistance to chloramphenicol and *dfrA-18 *gene that also confers resistance to trimethoprim was done as described previously [7,12]. Genomic DNA from *V*. *cholerae *O139 strains ATCC 51394, CO594 and VO143 were used as positive controls templates for the screening of *sulII*, *dfr18, strB *and SXT respectively and that from O1 biotype El Tor strains KO194 was used for the screening for the *dfrA1 *gene.

### Detection of mobile genetic elements

All strains were further tested for the presence of the 3'-conserved sequence (3-CS) of integron class 1 using the forward primer targeting the *qacEΔ1 *and the reverse primer of the *sulI*1 gene encoding resistance to quaternary ammonium compounds (detergents) and sulphonamides, respectively. The gene cassettes flanked by the 5'-CS and the 3'-CS were amplified using a combination of primers that target the 3'-CS and the 5'-CS of the integron class 1. Primers and PCR conditions for the detection of *intI*1 gene belonging to integron class 1 was done as described previously by Rivera *et al*. [29] while detection of the 3'-CS and the variable cassette region was done as described previously by Dalsgaard *et al*. [30]. Detection of *intI2 *was performed as previously described by Falbo *et al*. [31]. Screening for the integrase specific to integron class 3 (*intI*3) and integron class 4 (*intI*4) was performed as detailed previously by Machado *et al*. and Shi *et al*. respectively [32,33].

We also conducted PCR experiments using the genomic DNA isolated from donors and transconjugants to verify the transfer of the *Tn*21 and the SXT/R391-like element. Detection of *Tn*21 transposon was done using *trpM*-specific primers and PCR conditions published previously by Villa *et al*. [34] while detection of *Tn*7 was done using PCR conditions and primers described previously by Hansson *et al*. [26]. The presence of the ICE was detected using primers for amplification of a 1035 bp fragment of the integrase gene specific for the SXT/R391-like element as described previously by Bhanumathi *et al*. [35]. Integration of the ICE into the chromosome was demonstrated by amplification of a PCR product of 825 bp corresponding to the right junction between the *attP *element of the ICE and the *prfC *chromosomal gene of the bacteria. Primers and PCR conditions used are similar to those published before by Pugliese *et al*. [7]. Strains from our culture collection known to harbour various genes of interest were used as appropriate positive controls in corresponding PCR experiments.

### Analysis of *Vibrio cholerae *virulence genes

All strains were screened for the presence of genes encoding virulence determinants in *V. cholerae *including cholera toxin (*ctxA*), zonula occludens toxin (*zot*), accessory cholera enterotoxin (*ace*), hemolysin (*hlyA*), and NAG-specific heat-stable toxin (*st*). Detection of the *tcpA *gene specific to the El Tor and Classical biotypes was done using a common forward primer and biotype-specific reverse primers. Similarly, two forward primers were used for the detection of the biotype-specific haemolysin gene (*hylA*). PCR conditions and primers used for the detection of *tcpA, ompU, tcpI*, *toxR *and *hylA *genes were similar to those described previously by Rivera *et al*. [29] while detection of the *ctxA *gene was done using primers and conditions described before by Fields *et al*. [36]. Genomic DNA from *V. cholerae *O139 strain ATCC 51394 was used as a positive control in screening for *ctxA, zot, ace, tcpA, ompU, tcpI*, and *toxR *genes. For detection of the four *rstR *gene alleles, a single reverse primer was used in combination with forward primers specific for each of the four *rstR *gene alleles as described previously by Nusrin *et al*. [37].

### Plasmid analysis

DNA for plasmid analysis was extracted using the method of Kado and Liu [38] with a few modifications [39]. DNA was resuspended in 50 μl of TE buffer containing 10 mM Tris, and 1 mM EDTA (pH 8) and separated by electrophoresis on 0.8% agarose gel for 4 hours at 4 Volt/cm. Plasmids of known sizes isolated from *E. coli *V517 and 39R861 were used as controls.

### PFGE

Selection of strains used for genotypic analysis was based on location and year of isolation and antibiotic resistance profiles. PFGE was performed using the Pulsenet recommended procedure [40]. The plugs containing agarose-embedded DNA were digested with 50 Units of *Sfi*I (40 Units/μL) or *Not*I (10 Units/μL). Fragments from *Xba*1-digested *Salmonella *Braenderup H9812 were used as molecular size markers.

## Results and discussion

### Antibiotic susceptibility tests and conjugation tests

All the 65 strains showing resistance to the Chl-Strep-Sul-Trim combinations transferred this phenotype to *E. coli *C600 *en bloc*. The frequency of transfer, expressed as number of transconjugants per recipients, ranged between 2.3 × 10^-6 ^and 3.0 × 10^-6 ^with an average of 2.6 × 10^-6^. PCR analysis of the donor strains and the *E. coli *C600 transconjugants amplified a 626 bp fragment of *sulII *gene encoding resistance to sulfamethoxazole, a 278 bp amplicon corresponding to the *dfrA1 *gene encoding resistance to trimethoprim, a 515 bp fragment of *strB *encoding resistance to streptomycin, a 526 bp fragment of *floR *gene conferring resistance to chloramphenicol and a 1035 bp fragment corresponding to the integrase gene of the SXT/R391 ICE family, thus confirming co-transfer of resistance markers and this element. The *trpM *gene of the transposon *Tn*21 was not detected in the transconjugants indicating that this transposon had not been acquired via conjugation. The *dfrA18 *gen*e *was not detected in any of the isolates analysed. Similarly, attempts to isolate plasmids in the donor strains and transconjugants were not successful. These results are in agreement with those obtained by Pugliese *et al*. [7] who demonstrated the co-transfer of the SXT-like element with the genes encoding the Chl-Strep-Sul-Trim phenotype in O1 strains isolated locally in during the 1998-1999. These workers also found that some strains had an incC plasmid harbouring a gene conferring resistance to tetracycline and while other strains were resistant to ampicillin but we did not identify any strain in our collection bearing these resistance patterns. *V. cholerae *O1 strains resistant to tetracycline have previously been reported in Kenya [6] and Zambia [41] in the 1990s, but those isolated from Ethiopia [42] and Somalia [43] in the same period were susceptible to this antibiotic. Furthermore, strains isolated from previous outbreaks in Kenya were known to exhibit resistance to ampicillin [7], doxycycline and streptomycin [44]. None of the strains we studied were resistant to furazolidone as was the case with strains isolated from Mozambican immigrants in South Africa [45]. Similarly, none of these strains were resistant to ceftriaxone, cefotaxime, nalidixic acid, amikacin and gentamicin as has been the case with strains previously reported from Ghana [46]. All the strains we analysed also lacked the *dfrA18 *gene encoding resistance to trimethoprim in O1 and non-O1 strains isolated in India [47].

Genetic and environmental factors that may be responsible for the apparent serotype shift from Ogawa to Inaba in recent outbreaks in Kenya remain to be elucidated. While strains that do not harbour the SXT/R391-like element and those bearing the incC plasmids were not available for analysis alongside those included in our study, it is apparent that the gradual emergence of a population of *V. cholerae *O1 strains bearing the SXT/R391-like element as a major cause of cholera outbreaks in Kenya has occurred independent of antibiotic resistance acquisition.

It remains to be determined exactly when the SXT/R391-like ICE emerged in pathogenic *V. cholera *strains in Kenya because isolates obtained locally between 1975 and 1983 were known to exhibit resistance to antibiotics encountered in the Chl-Strep-Sul-Trim phenotype [5,6] that has lately been associated to the presence of the SXT-type ICEs [12]. Although it is well established that cholera came to Africa from Asia in the 1970s, it is only suspected that the SXT-like elements have been present in African *Vibrio *spp even before the emergence of the *V. cholerae *O139 from which the first SXT element, SXT^MO10^, was identified [12]. ICE-like elements have been detected in O1 clinical strains isolated in 1992 in Angola and *V. parahaemolyticus *clinical strains from the same country isolated in 1991 were also shown to contain SXT-related ICEs that do not mediate resistance to antibiotics [14]. Similarly, analysis of O1 El Tor clinical isolates from Algeria isolated in 1994 suggests the presence of SXT-like ICEs mediating trimethoprim resistance [48]. However, the isolates from the 1994 outbreak in the Goma refugee camp in Zaire did not harbour this element [13]. Our study demonstrates that the O1 El Tor strains bearing the SXT/R391-like ICE were in circulation in Kenya in the 1994-1996 period and have continued to persist in recent outbreaks. This may suggest that the 6 strains isolated from the two outbreaks in 1994-1996 in Kwale, a coastal town of Kenya, are some of the oldest strains in the region known to harbour this integrating conjugative element in this part of the continent.

### Analysis for mobile genetic elements and *Vibrio cholerae *Pathogenicity Island

All the 65 O1 strains were positive for all the *V. cholerae *pathogenic genes except for the NAG-specific heat-stable toxin (*st*). These strains were also positive for the *IntI*4 integrase belonging to integron class 4, asuper-integron believed to be important in shuffling the *Vibrio cholerae *genome [25]. It is worth noting that the *st *gene normally occurs as a cassette (*sto*) within *Int*4 region in some *V. cholerae *strains but not in others [26]. Besides the *st *gene, another pathogenicity determinant, *mrhA*, is frequently detected in SI region of O1 and non-O1strains [49]. It is not clear whether the presence of the *st *gene improves biological fitness of the host but appears to be dispensable without compromising the ability to cause disease in pathogenic strains.

Besides the SXT elements, other mobile genetic elements implicated in the spread of antibiotic resistance phenotype in *V. cholerae *from Africa include conjugative plasmids belonging to class C [5,7], integron class 1 [41,46], and integron class 2 [41]. Although the isolates we studied carried the SXT element, they lacked the class 1, 2, and 3 integrons and did not harbour any conjugative plasmids. All the strains were negative for the transposase gene belonging to *Tn*7 but were positive for the *trpM *gene associated with *Tn*21. The *Tn*7 has frequently been detected in gram negative strains containing integron class 2 [26]. On the other hand, *Tn*21 and its relatives are major agents in the dissemination of mercury resistance and antibiotic resistance genes in gram negative bacteria but not all *Tn*21-like transposons are associated with antibiotic resistance and there are variations in the diversity of antibiotic resistance genes detected in *Tn*21-like transposons that harbour antibiotic resistance markers [50]. PCR analysis of transconjugants did not detect the *Tn*21 implying that this transposon was not co-transferred with the SXT/R391-like element during conjugation. We were however not able to determine if this element confers mercury resistance to the strains we studied or if it is physically linked to any antibiotic resistance markers. It is also not clear if this transposon has all the other genes responsible for transposition such as *tnpA*, *tnpR*, *res*, and inverted repeats or if it exists as a defective transposon in these strains. However, the presence of the *trpM *gene suggests that although the strains carrying the SXT/R391-like elements lack multiple resistant integrons, this transposon is genetically ready to accept such elements because integrons are normally located adjacent to this gene [50]. It has been suggested that *Tn*21-like transposons which confer multiple antibiotic resistance descended from an ancestral mercury resistance transposon like *Tn*501 by successive insertions of antibiotic resistances and/or insertion sequences [51]. It is therefore important to further characterize *Tn*21 in pathogenic *V. cholerae *strains.

All the 65 strains were positive for the CTX^ET^Φ but negative for all the other *CTX*Φ phage repressor gene alleles and this contradicts with the study on O1 El Tor strains isolates from Mozambique [52] and India [20] which have been reported to harbour the CTX^class^Φ repressor. Such El Tor Strains carrying the CTX^class^Φ repressor are now designated as the Matlab variants of *V. cholerae *[53]. Our finding on the diversity of the CTX^ET^Φ repressor and the absence of the other *rstR *genes in all the strains further indicate the need for detailed studies on the genetic diversity of *V. cholerae *strains from different parts of the continent to gain insight into the evolutionary trends of *V. cholerae *species causing epidemics in Africa.

### Pulsed Field Gel Electrophoresis

**Figure 2:**
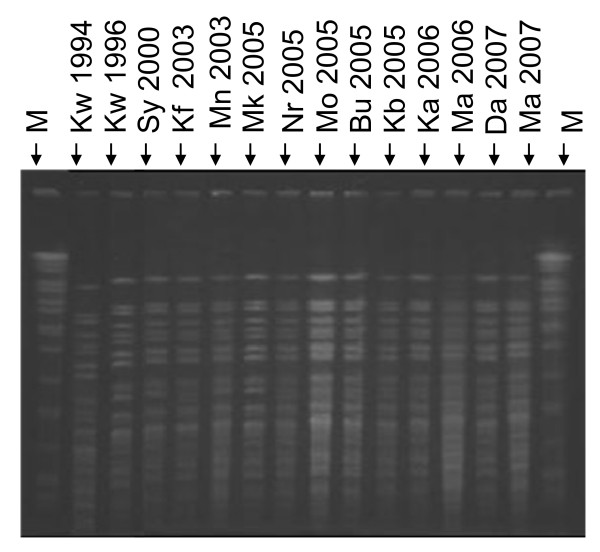
**PFGE of Not1 digested genomic DNA of *V*. Inaba strains isolated from various regions of Kenya between 1994 and 2007**. Genomic DNA from representative strains was digested with *Not*1 restriction enzyme and loaded as follows; **M**: molecular weight marker (*S*. Braenderup), **Kw**: Kwale, **Sy**: Siaya, **Mn**: Malindi, **Mk**: Makindu, **Nr**: Nairobi, **Kb**: Kibwezi, **Mo**: Mombasa, **Bu**: Busia, **Kf**: Kilifi, **Ka**: Kakuma, **Da**: Daadab, **Ma**; Mandera. The year when each of the isolate included in this experiment are also indicated.

Analysis of macro-restricted DNA using *Not*1 demonstrated that all the SXT-carrying strains produced indistinguishable digest patterns regardless of the year and region of isolation (Figure 2). Similarly, restriction digest analysis using *Sfi*1 showed that all strains were clonal (data not shown). The fact that all our strains showed identical pattern in antibiotic susceptibility patterns, pathogenicity genes, and the diversity of mobile genetic elements strongly suggest that this population of O1 strains that have caused outbreaks since 1994 to as recent as 2007 are clonally related. The absence of the *st *gene (which is common among non-01 and non-0139 strains) [19] and the absence of the classical biotype-specific *tcpA *and *hylA *genes in these strains further indicates that genetic exchanges between this population and other *V. cholerae *serotypes that might be in circulation in Kenya have been highly restricted. In a previous study by Jiang *et al*. [54] it was noted that a number of O1 strains from Kenya failed to cluster with those isolated from other parts of the world when using Amplified Fragment Length Polymorphism (AFLP) genotyping technique. Similarly, the study by Pugliese *et al*. [7] showed that strains that carried the SXT-element alone or in combination with an incC plasmid belonged to a unique RAPD cluster IV. In the same study [7], strains without this ICE were shown to belong to other cluster types shared by isolates from Ethiopia and Somali. It is also interesting to note that none of the isolates from 1998-1999 study shared a RAPD cluster with strains isolated in India and Bahrain isolated in 1948 and 1978. Such observations have led to a theory that some toxigenic *V. cholerae *strains circulating in different countries may not have originated from a single clone in Asia as is popularly believed, but may have been derived locally from genetic exchange between the Asian O1 strains and the O1 or non-O1 strains from local environments [54].

## Conclusions

We observed that antibiotic susceptibility and genomic content of the strains bearing the SXT/R391-like ICE that have been in circulation in Kenya between 1994 and 2007 has not changed significantly and there are indications that these strains have undergone minimum genotypic changes during this entire period. In the absence of older isolates for molecular characterization, it is not possible to determine whether other clones of *V. cholerae *bearing this ICE have existed in Kenya and is also difficult to determine when the SXT/R391-bearing clone emerged as the major cause of outbreaks in Kenya. In order to understand the epidemiological trends of cholera outbreaks in the region, there is need for further studies to determine evolutionary trends among strains isolated from the African region and compare them with those from other parts of the world.

## Authors' contributions

JNK designed and coordinated the study, carried out molecular characterization studies and drafted the manuscript. SMK participated in the design and supervision of the study and revision of the manuscript. BMG revised the manuscript and supervised the study. NCW participated in manuscript revision. SMS provided strains from earlier outbreaks and revised the manuscript. PB participated in the study design, supervision of molecular characterization studies in Belgium and revision of manuscript. All authors read and approved the final manuscript.

## Authors' information

JNK is a research Scientist at the Kenya Medical Research Institute (KEMRI) and doctoral fellow at Katholieke Universiteit Leuven, Belgium. He holds an MSc (Microbiology) and MSc (Molecular Biology, K.U.Leuven) where he is currently pursuing a PhD in Bioscience Engineering at the Department of Biosystems. His work is supported by a scholarship from the Vlaamse Interuniversitaire Raad (VLIR), Belgium. SMK, NCW and SMS are Scientists at KEMRI, Kenya. SMK is a Wellcome Trust Research fellow and an opinion leader in the field of antibiotic resistance in the East African region while NCW is the former Director Centre for Microbiology Research, KEMRI. BMG is Professor of immunology at the K.U.Leuven (Faculty of Bioscience Engineering) and the University of Ghent (UGent, Faculty of Veterinary Medicine), Belgium while PB is a Senior Research Scientist at the Veterinary and Agrochemical Research Centre (VAR) and an expert in the field of antibiotic resistance in Belgium. He is also a Professor at the Faculty of Veterinary Medicine at UGent.

## References

[B1] World Health Organization Global Task Force on Cholera Control. Cholera country profile: Kenya http://www.who.int/cholera/countries/KenyaCountryProfileMay2008.pdf

[B2] World Health Organization Cholera, 1998 Wkly Epidemiol Rec 1999 74 257 264 10453697

[B3] World Health Organization Cholera, 1999 Wkly Epidemiol Rec 2000 75 249 256 10953668

[B4] Mugoya I Kariuki S Galgalo T Njuguna C Omollo J Njoroge J Kalani R Nzioka C Tetteh C Bedno S Breiman RF Feikin DR Rapid spread of *Vibrio cholerae *O1 throughout Kenya, 2005 Am J Trop Med Hyg 2008 78 527 533 18337355

[B5] Iwanaga M Mori K Kaviti JN *Vibrio cholerae *O1 isolated in Kenya J Clin Microbiol 1982 16 4 742 743 7153323 10.1128/jcm.16.4.742-743.1982 PMC272459

[B6] Ichinose Y Ehara M Watanabe S Shimodori S Waiyaki PG Kibue AM Sang FC Ngugi J Kaviti JN The characterization of *Vibrio cholerae *isolated in Kenya in 1983 J Trop Med Hyg 1986 89 269 276 3795327

[B7] Pugliese N Maimone F Scrascia M Materu SF Pazzani C SXT-related integrating conjugative element and IncC plasmids in *Vibrio cholerae *O1 strains in Eastern Africa J Antimicrob Chemother 2009 63 3 438 442 10.1093/jac/dkn542 19155226

[B8] Beaber JW Burrus V Hochhut B Waldor MK Comparison of SXT and R391, two conjugative integrating elements: definition of a genetic backbone for the mobilization of resistance determinants Cell MolLife Sci 2002 59 12 2065 2070 10.1007/s000180200006 PMC11146103 12568332

[B9] Nunes-Düby SE Kwon HJ Tirumalai RS Ellenberger T Landy A Similarities and differences among 105 members of the Int family of site-specific recombinases Nucleic Acids Res 1998 26 2 391 406 10.1093/nar/26.2.391 9421491 PMC147275

[B10] Hochhut B Waldor MK Site-specific integration of the conjugal *Vibrio cholerae *SXT element into prfC Mol Microbiol 1999 32 1 99 110 10.1046/j.1365-2958.1999.01330.x 10216863

[B11] Hochhut B Beaber JW Woodgate R Waldor MK Formation of chromosomal tandem arrays of the SXT element and R391, two conjugative chromosomally integrating elements that share an attachment site J Bacteriol 2001 183 4 1124 1132 10.1128/JB.183.4.1124-1132.2001 11157923 PMC94984

[B12] Hochhut B Lotfi Y Mazel D Faruque SM Woodgate R Waldor MK Molecular analysis of antibiotic resistance gene clusters in *Vibrio cholerae *O139 and O1 SXT constins Antimicrob Agents Chemother 2001 45 11 2991 3000 10.1128/AAC.45.11.2991-3000.2001 11600347 PMC90773

[B13] Waldor MK Tschäpe H Mekalanos JJ A new type of conjugative transposon encodes resistance to sulfamethoxazole, trimethoprim, and streptomycin in *Vibrio cholerae *O139 J Bacteriol 1996 178 14 4157 4165 8763944 10.1128/jb.178.14.4157-4165.1996 PMC178173

[B14] Burrus V Marrero J Waldor MK The current ICE age: biology and evolution of SXT-related integrating conjugative element Plasmid 2006 55 3 173 183 10.1016/j.plasmid.2006.01.001 16530834

[B15] Kimsey HH Waldor MK CTXphi immunity: application in the development of cholera vaccines Proc Natl Acad Sci USA 1998 95 12 7035 7039 10.1073/pnas.95.12.7035 9618534 PMC22729

[B16] Davis BM Moyer KE Boyd EF Waldor MK CTX prophages in classical biotype *Vibrio cholerae *: functional phage genes but dysfunctional phage genomes J Bacteriol 2000 182 24 6992 6998 10.1128/JB.182.24.6992-6998.2000 11092860 PMC94825

[B17] Davis BM Kimsey HH Chang W Waldor MK The *Vibrio cholerae *O139 Calcutta bacteriophage CTXphi is infectious and encodes a novel repressor J Bacteriol 1999 181 21 6779 6787 10542181 10.1128/jb.181.21.6779-6787.1999 PMC94144

[B18] Mukhopadhyay AK Chakraborty S Takeda Y Nair GB Berg DE Characterization of VPI pathogenicity island and CTXphi prophage in environmental strains of *Vibrio cholerae* JBacteriol 2001 183 16 4737 4746 10.1128/JB.183.16.4737-4746.2001 PMC99527 11466276

[B19] Nair GB Faruque SM Bhuiyan NA Kamruzzaman M Siddique AK Sack DA New variants of *Vibrio cholerae *O1 biotype El Tor with attributes of the classical biotype from hospitalized patients with acute diarrhea in Bangladesh J Clin Microbiol 2002 40 9 3296 3299 12202569 10.1128/JCM.40.9.3296-3299.2002 PMC130785

[B20] Chatterjee S Patra T Ghosh K Raychoudhuri A Pazhani GP Das M Sarkar B Bhadra RK Mukhopadhyay AK Takeda Y Nair GB Ramamurthy T Nandy RK *Vibrio cholerae *O1 clinical strains isolated in 1992 in Kolkata with progenitor traits of the 2004 Mozambique variant J Med Microbiol 2009 58 2 239 247 10.1099/jmm.0.003780-0 19141743

[B21] Faruque SM Tam VC Chowdhury N Diraphat P Dziejman M Heidelberg JF Clemens JD Mekalanos JJ Nair GB Genomic analysis of the Mozambique strain of *Vibrio cholerae *O1 reveals the origin of El Tor strains carrying classical CTX prophage Proc Natl Acad Sci USA 2007 104 12 5151 5156 10.1073/pnas.0700365104 17360342 PMC1829278

[B22] Hall RM Collis CM Antibiotic resistance in gram-negative bacteria: the role of gene cassettes and integrons DrugResist Updat 1998 1 2 109 119 10.1016/S1368-7646(98)80026-5 16904397

[B23] Rowe-Magnus DA Mazel D The role of integrons in antibiotic resistance gene capture Int J Med Microbiol 2002 292 2 115 125 10.1078/1438-4221-00197 12195734

[B24] Rowe-Magnus DA Mazel D Resistance gene capture Curr Opin Microbiol 1999 2 5 483 488 10.1016/S1369-5274(99)00004-1 10508722

[B25] Rowe-Magnus DA Guérout AM Mazel D Super-integrons Res Microbiol 1999 150 9-10 641 651 10.1016/S0923-2508(99)00127-8 10673003

[B26] Hansson K Sundström L Pelletier A Roy PH IntI2 integron integrase in Tn7 J Bacteriol 2002 184 6 1712 1721 10.1128/JB.184.6.1712-1721.2002 11872723 PMC134885

[B27] Clinical and Laboratory Standards Institute Performance standards for antimicrobial susceptibility testing. M100-S17 2007 CLSI, Wayne, PA 10.1128/JCM.00213-21 PMC8601225 34550809

[B28] Ramachandran D Bhanumathi R Singh DV Multiplex PCR for detection of antibiotic resistance genes and the SXT element: application in the characterization of *Vibrio cholerae* J Med Microbiol 2007 56 3 346 351 10.1099/jmm.0.46655-0 17314365

[B29] Rivera IN Chun J Huq A Sack RB Colwell RR Genotypes Associated with Virulence in Environmental Isolates of *Vibrio cholerae* Appl Environ Microbiol 2001 67 2421 2429 10.1128/AEM.67.6.2421-2429.2001 11375146 PMC92890

[B30] Dalsgaard A Forslund A Serichantalergs O Sandvang D Distribution and content of class 1 integrons in different *Vibrio cholerae *O-serotype strains isolated in Thailand Antimicrob Agents Chemother 2000 44 5 1315 1321 10.1128/AAC.44.5.1315-1321.2000 10770768 PMC89861

[B31] Falbo V Carattoli A Tosini F Pezzella C Dionisi AM Luzzi I Antibiotic resistance conferred by a conjugative plasmid and a class I integron in *Vibrio cholerae *O1 El Tor strains isolated in Albania and Italy Antimicrob Agents Chemother 1999 43 3 693 696 10049292 10.1128/aac.43.3.693 PMC89185

[B32] Machado E Cantón R Baquero F Galán JC Rollán A Peixe L Coque TM Integron content of extended-spectrum-beta-lactamase-producing *Escherichia coli *strains over 12 years in a single hospital in Madrid, Spain Antimicrob AgentsChemother 2005 49 5 1823 1829 10.1128/AAC.49.5.1823-1829.2005 PMC1087637 15855502

[B33] Shi L Fujihara K Sato T Ito H Garg P Chakrabarty R Ramamurthy T Nair GB Takeda Y Yamasaki SV Distribution and characterization of integrons in various serogroups of *Vibrio cholerae *strains isolated from diarrhoeal patients between 1992 and 2000 in Kolkata, India J Med Microbiol 2006 55 5 575 583 10.1099/jmm.0.46339-0 16585645

[B34] Villa L Visca P Tosini F Pezzella C Carattoli A Composite integron array generated by insertion of an ORF341-type integron within a Tn21-like element Microb Drug Resist 2002 8 1 1 8 10.1089/10766290252913692 12002644

[B35] Bhanumathi R Sabeena F Isac SR Shukla BN Singh DV Molecular characterization of *Vibrio cholerae *O139 bengal isolated from water and the aquatic plant *Eichhornia crassipes *in the River Ganga, Varanasi, India Appl Environ Microbiol 2003 69 4 2389 2394 10.1128/AEM.69.4.2389-2394.2003 12676727 PMC154771

[B36] Fields PI Popovic T Wachsmuth K Olsvik O Use of polymerase chain reaction for detection of toxigenic *Vibrio cholerae *O1 strains from the Latin American cholera epidemic J Clin Microbiol 1992 30 8 2118 2121 1500520 10.1128/jcm.30.8.2118-2121.1992 PMC265454

[B37] Nusrin S Khan GY Bhuiyan NA Ansaruzzaman M Hossain MA Safa A Khan R Faruque SM Sack DA Hamabata T Takeda Y Nair GB Diverse CTX phages among toxigenic *Vibrio cholerae *O1 and O139 strains isolated between 1994 and 2002 in an area where cholera is endemic in Bangladesh J Clin Microbiol 2004 42 12 5854 5856 10.1128/JCM.42.12.5854-5856.2004 15583324 PMC535256

[B38] Kado CI Liu ST Rapid procedure for detection and isolation of large and small plasmids J Bacteriol 1981 145 3 1365 1373 7009583 10.1128/jb.145.3.1365-1373.1981 PMC217141

[B39] Goldstein C Lee MD Sanchez S Hudson C Phillips B Register B Grady M Liebert C Summers AO White DG Maurer JJ Incidence of class 1 and 2 integrases in clinical and commensal bacteria from livestock, companion animals, and exotics Antimicrob Agents Chemother 2001 45 3 723 726 10.1128/AAC.45.3.723-726.2001 11181350 PMC90363

[B40] Cooper KL Luey CK Bird M Terajima J Nair GB Kam KM Arakawa E Safa A Cheung DT Law CP Watanabe H Kubota K Swaminathan B Ribot EM Development and validation of a PulseNet standardized pulsed-field gel electrophoresis protocol for subtyping of *Vibrio cholerae* Foodborne Pathog Dis 2006 3 1 51 58 10.1089/fpd.2006.3.51 16602979

[B41] Mwansa JC Mwaba J Lukwesa C Bhuiyan NA Ansaruzzaman M Ramamurthy T Alam M Balakrish Nair G Multiply antibiotic-resistant *Vibrio cholerae *O1 biotype El Tor strains emerge during cholera outbreaks in Zambia Epidemiol Infect 2007 135 5 847 853 10.1017/S0950268806007254 17121691 PMC2870619

[B42] Scrascia M Pugliese N Maimone F Mohamud KA Ali IA Grimont PA Pazzani C Cholera in Ethiopia in the 1990s: epidemiologic patterns, clonal analysis, and antimicrobial resistance Int J Med Microbiol 2009 299 5 367 372 10.1016/j.ijmm.2008.10.004 19121605

[B43] Scrascia M Pugliese N Maimone F Mohamud KA Grimont PA Materu SF Pazzani C Clonal relationship among *Vibrio cholerae *O1 El Tor strains isolated in Somalia Int J Med Microbiol 2009 299 3 203 207 10.1016/j.ijmm.2008.07.003 18774337

[B44] Scrascia M Maimone F Mohamud KA Materu SF Grimont F Grimont PA Pazzani C Clonal relationship among *Vibrio cholerae *O1 El Tor strains causing the largest cholera epidemic in Kenya in the late 1990s J Clin Microbiol 2006 44 9 3401 3404 10.1128/JCM.00611-06 16954285 PMC1594678

[B45] Dalsgaard A Forslund A Sandvang D Arntzen L Keddy K *Vibrio cholera *e O1 outbreak isolates in Mozambique and South Africa in 1998 are multiple-drug resistant, contain the SXT element and the aadA2 gene located on class 1 integrons J Antimicrob Chemother 2001 48 6 827 838 10.1093/jac/48.6.827 11733467

[B46] Opintan JA Newman MJ Nsiah-Poodoh OA Okeke IN *Vibrio cholerae *O1 from Accra, Ghana carrying a class 2 integron and the SXT element J Antimicrob Chemother 2008 62 5 929 933 10.1093/jac/dkn334 18755696 PMC2566517

[B47] Mohapatra H Mohapatra SS Mantri CK Colwell RR Singh DV *Vibrio cholerae *non-O1, non-O139 strains isolated before 1992 from Varanasi, India are multiple drug resistant, contain intSXT, dfr18 and aadA5 genes Environ Microbiol 2008 10 4 866 873 10.1111/j.1462-2920.2007.01502.x 18201198

[B48] Korichi MN Belhocine S Rahal K Inc J plasmids identified for the first time in *Vibrio cholerae *El Tor Med Trop (Mars) 1997 57 3 249 252 9513150

[B49] vanDongen WMAM Vlerken V Degraaf FK Nucleotide sequence of a DNA fragment encoding a *Vibrio cholerae *haemagglutinin Mol Gen (Life Sci Adv) 1987 6 85 91

[B50] Liebert CA Hall RM Summers AO Transposon Tn21, flagship of the floating genome Microbiol Mol Biol Rev 1999 63 3 507 522 10477306 10.1128/mmbr.63.3.507-522.1999 PMC103744

[B51] Tanaka M Yamamoto T Sawai T Evolution of complex resistance transposons from an ancestral mercury transposon J Bacteriol 1983 153 3 1432 1438 6298184 10.1128/jb.153.3.1432-1438.1983 PMC221794

[B52] Ansaruzzaman M Bhuiyan NA Nair BG Sack DA Lucas M Deen JL Ampuero J Chaignat CL Mozambique Cholera vaccine Demonstration Project Coordination Group Cholera in Mozambique, variant of *Vibrio cholerae* Emerg Infect Dis 2004 10 11 2057 2059 16010751 10.3201/eid1011.040682 PMC3329043

[B53] Safa A Bhuyian NA Nusrin S Ansaruzzaman M Alam M Hamabata T Takeda Y Sack DA Nair GB Genetic characteristics of Matlab variants of *Vibrio cholerae *O1 that are hybrids between classical and El Tor biotypes J Med Microbiol 2006 55 11 1563 1569 10.1099/jmm.0.46689-0 17030917

[B54] Jiang SC Matte M Matte G Huq A Colwell RR Genetic diversity of clinical and environmental isolates of *Vibrio cholerae *determined by amplified fragment length polymorphism fingerprinting Appl Environ Microbiol 2000 66 1 148 153 10.1128/AEM.66.1.148-153.2000 10618216 PMC91798

